# Role of cAMP Signaling in the Survival and Infectivity of the Protozoan Parasite, *Leishmania donovani*

**DOI:** 10.4061/2011/782971

**Published:** 2011-06-05

**Authors:** Arunima Biswas, Arijit Bhattacharya, Pijush K. Das

**Affiliations:** ^1^Molecular Cell Biology Laboratory, Infectious Diseases and Immunology Division, Indian Institute of Chemical Biology, Kolkata 700032, India; ^2^Department of Biotechnology, Presidency College, Kolkata 700073, India

## Abstract

*Leishmania donovani*, while invading macrophages, encounters striking shift in temperature and pH (from 22°C and pH 7.2 to 37°C and pH 5.5), which act as the key environmental trigger for differentiation, and increases cAMP level and cAMP-mediated responses. For comprehensive understanding of cAMP signaling, we studied the enzymes related to cAMP metabolism. A stage-specific and developmentally regulated isoform of receptor adenylate cyclase (LdRACA) showed to regulate differentiation-coupled induction of cAMP. The soluble acidocalcisomal pyrophosphatase, Ldvsp1, was the major isoform regulating cAMP level in association with LdRACA. A differentially expressed soluble cytosolic cAMP phosphodiesterase (LdPDEA) might be related to infection establishment by shifting trypanothione pool utilization bias toward antioxidant defense. We identified and cloned a functional cAMP-binding effector molecule from *L. donovani* (a regulatory subunit of cAMP-dependent protein kinase, LdPKAR) that may modulate metacyclogenesis through induction of autophagy. This study reveals the significance of cAMP signaling in parasite survival and infectivity.

## 1. Introduction

Infection by protozoan parasites of the genus *Leishmania* results in a spectrum of clinical manifestations referred to collectively as leishmaniases. The clinical manifestations range in severity from spontaneously healing cutaneous ulcers by *L. major* infection to potentially fatal visceral disease by *L. donovani* infection. The parasite is a digenic one and in its infective cycle, the parasite is transmitted as promastigote from the gut of insect vector female phlebotomine flies to mammalian hosts. The procyclic promastigotes get converted to metacyclic ones and are phagocytosed by mammalian macrophages where they convert into amastigote form, which is able to survive, and replicate within the phagolysosome. Along with a substantial alteration of nutrient availability, the parasite must adapt to new conditions of temperature and pH (37°C and pH 5.5) which acts as an initial environmental stress to the parasite. This physical conditioning has proved indispensable for *Leishmania* differentiation and *in vitro* transformation protocols are already in use mimicking the physical condition encountered in mammalian host [1, 2]. After their phagocytosis by macrophages at the initial stages of infection the parasites suffer another stress caused by the respiratory burst of macrophages, its first line of defense, producing reactive oxygen and reactive nitrogen species [3, 4]. Macrophages also produce different cytokines and chemokines that regulate their activity as well as regulate the recruitment and activation of other inflammatory cells. Cell-mediated immunity, which depends on the differentiation of Th0 cells to Th1 cells is also regulated by macrophage functions. IL-12 and IFN-*γ* secreted by activated macrophages play important role in differentiation of naïve T-helper cells into proinflammatory Th1 subset. However, there are some intracellular parasites like *Leishmania* that are able to impair these activities by taking advantage of the host anti-inflammatory response to avoid self-damage by modulating its own biology and host environment to persist successfully inside the host. Even in the face of exposure to toxic prooxidants a subset of the *Leishmania* parasites that invades the host macrophages survives and subsequently converts into intracellular amastigotes, finally leading to disease manifestation [5]. But the molecular mechanism by which the parasite circumvents the toxic effects of these reactive oxygen and nitrogen species is yet to be deciphered. 

 Few previous studies suggested that in *Leishmania,* genes like superoxide dismutase, peroxidoxin, and trypanothione reductase are implicated in antioxidant defense against reactive oxygen species (ROS) and reactive nitrogen intermediates (RNI) [6–8]. Disruption of these genes or transfection with transdominant inactive counterpart renders parasites more susceptible to intracellular killing in macrophages capable of generating reactive oxygen intermediates [6–10]. But, in *Leishmania*, in the absence of any known transcription factors, it is really unknown what triggers the expressions of the genes speculated to be associated with its antioxidant system. Interestingly, like in many lower organisms, environmental cues seemed to play some important roles in controlling biology of the parasite. Preexposure to environmental stress (pH 5.5 and temperature 37°C) has been shown to induce resistance against oxidative damage in this organism [3, 11]. The ability of *Leishmania* parasites to resist oxidative damage was observed to be coupled with their transformation to amastigote stage and there may be more than one mechanism of environmental sensing along with stress exposure, which finally trigger differentiation of the parasite. cAMP response has been implicated as one of the major environmental sensing machineries associated with stress response in many unicellular eukaryotes like *Plasmodium*, *Trypanosoma, *and others. cAMP in malarial parasite, *Plasmodium falciparum* triggers the conversion of asexual erythrocytic ring-stage parasites to sexual precursors, gametocytes [12]. *P. falciparum* could synthesize its own cAMP by adenylate cyclase (AC) which is uniquely not stimulated by mammalian AC activator Forskolin or heteromeric G-protein activators AIF_4_. Moreover, cAMP signaling effector molecule Protein Kinase A (PKA) plays an important role in conductance of anions across the host cell membrane of *Plasmodium*-infected RBC [13]. It is now known that PKAR (PKA regulatory subunit) may be involved in activation of anion conductance channel in *P. falciparum*-infected RBC [14]. Activation of PKC or cAMP-dependent signaling pathways in *Entamoeba histolytica* triggers the phosphorylation of proteins involved in actin rear-arrangements necessary for adhesion and locomotion. Moreover, cAMP-response elements could play an important role in regulating actin expression and organization in signaling processes activated during tissue invasion. cAMP also plays an important role in Trypanosome differentiation from long slender form to short stumpy form, the form in which the cAMP level declines [15]. Moreover, adenylate cyclase activity is stimulated by Ca^2+^, which seemed to have a receptor located in the membrane or as a part of AC. Also in *Plasmodium*, evidences identified intracellular Ca^2+^ store utilized by both melatonin and cAMP pathways. Also another component of cAMP signaling, the phosphodiesterases (PDEs) has transmembrane domains suggesting that they are integral membrane proteins. Hence, it was indeed necessary to study whether cAMP has similar importance in the parasite survival and infectivity. The Ras-cAMP pathway serves as a negative regulator of stress response in *Saccharomyces cerevisae* [16, 17]. 

**Figure 1:**
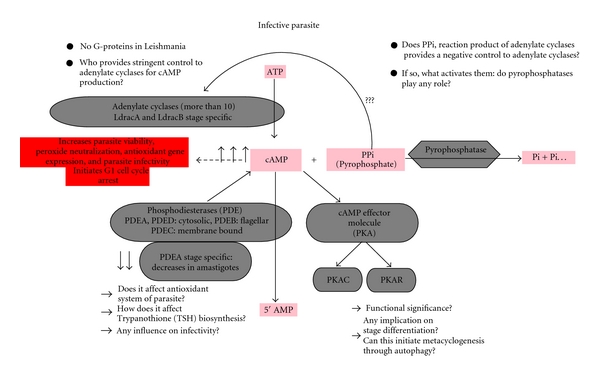
Enzymes intimately associated with cAMP metabolism in *Leishmania*. Cyclic adenosine monophosphate (cAMP) is formed from adenosine triphosphate (ATP) by adenylate cyclases where pyrophosphate (PPi) is also produced as one of reaction products which is hydrolyzed by pyrophosphatases to inorganic phosphate (Pi). Downstream to cAMP, leishmanial phosphodiesterases (PDE) hydrolyzes cAMP to 5′ adenosine monophosphate (5′AMP). There are 5 different PDEs in the parasite (PDEA, PDEB1, PDEB2, PDEC, and PDED). cAMP-dependent protein kinase A (PKA) exists as an inactive tetramer consisting of two catalytic subunits (PKAC) and two regulatory subunits (PKAR). Binding of cAMP to regulatory subunit releases catalytic subunit.

 This review will focus on developments in the field of *Leishmania* cAMP signaling and its control. We have tried to assess the functions of all the enzymes that are intimately associated with cAMP metabolism in the parasite (Figure 1). The multitudinous functions of cAMP require precise spatial and temporal control of its production, degradation, and detection. Though novel proteins have recently been identified that critically modulate cAMP signal in several organisms, not much is known about cAMP signaling in *Leishmania *(Figure 1). In this review, we sought to focus on the molecular mechanisms whereby *Leishmania *parasites can subvert host surveillance by activating its own antioxidant machineries by cAMP-mediated signaling. We would also like to shed some light on the mechanism of action of the leishmanial adenylyl cyclases towards the positive modulation of cAMP in the absence of canonical heteromeric G proteins and genes for G-protein-coupled receptors [18]. We focus on the action of cAMP on *Leishmania* lifecycle that helps its survival inside macrophages and sought to discuss the role of cAMP-dependent phosphodiesterases in modulating the cAMP signaling in the parasite.

## 2. Role of cAMP in *Leishmania* Survival and Infectivity

*Leishmania* thrives inside the gut of sand fly at a temperature of 22°C and pH 7.4 and encounters a huge shift in temperature and pH to 37°C and pH 5.5 when it invades mammalian macrophages where a subset of parasite survives the oxidative stress of the macrophages to get converted into amastigotes. The initial environmental stress in the macrophage environment induces differentiation of the parasites from promastigotes to amastigotes and it was deciphered by us that the differentiation condition (37°C and pH 5.5) increases the resistivity of the parasites against induced oxidative stress by H_2_O_2_ and peroxynitrite [19]. Moreover, it was also shown that such parasites could infect IFN-*γ*-activated macrophages with more efficiency than the parasites not exposed to differentiation condition [19]. Since differentiation condition is nothing but an environmental cue for the parasite, it was exigent to look for a molecule which could sense such cue to trigger a signaling cascade leading to parasite infectivity and survival within the macrophages. As cyclic nucleotides were known to be important modulators of environmental conditions and speculations were there regarding its role in kinetoplastidae differentiation, cAMP level in the parasite was checked after exposing them to differentiation condition. Results indicated striking elevation of cAMP level in such parasites within 1 hourour of stress exposure. Not only that, cAMP-dependent protein kinase activity (PKA) also increased simultaneously and the substrate level phosphorylation of the same was also elevated [19]. This indicated that cAMP might have an interesting role to play in leishmanial survival and infectivity.

### 2.1. cAMP Is an Environmental Sensor and Cytoprotector in *Leishmania*

Since cAMP plays a pivotal role in the differentiation, cell movement, and stress response in several organisms like *Dictyostellium* and *Trypanosoma*, more understanding was required regarding the role of cAMP in parasite survival in macrophage's hostile environment. Increasing intracellular cAMP level by cell permeable cAMP analog, pCPT-cAMP resulted in increased resistance against H_2_O_2_ and peroxynitrite. On the other hand, enhanced resistance by exposure to differentiation condition could be reversed by adenylate cyclase inhibitor, DDA (dideoxyadenosine), and PKA inhibitor, H89. To further ascertain the ability of cAMP in cytoprotection of the parasites against H_2_O_2_, three parameters were checked: DNA degradation, protein carbonylation, and ultrastructural analysis. The extent of DNA degradation and protein carbonylation by H_2_O_2_ was reduced in pCPT-cAMP-treated and differentiation condition-exposed cells, which got reversed by treatment with DDA, and H89 [19]. Similarly, ultrastructural integrity was retained more in pCPT-CMP-treated cells than in normal macrophages. These observations suggest that differentiation condition triggers cAMP response, which enhances resistance against oxidants.

### 2.2. Role of cAMP in Cell Cycle Blockage

Morphological transformation of promastigotes to amastigotes by exposure to 37°C and pH 5.5 occurs during cell cycle arrest at G1 phase [20]. As resistance against oxidative damage and transformation are coupled and because cell cycle arrest initiates differentiation, we studied the effects of cAMP modulation on cell cycle of *Leishmania*. Cell cycle was studied after intracellular cAMP concentration was modulated by treating the cells with pCPTcAMP, DDA and H89. pCPTcAMP caused a significant G1 phase arrest whereas treatment with DDA, and H89 decreased such arrested condition [19]. This data indicated the involvement of cAMP in G1 arrest of the parasite during transformation. But why such G1 arrest was required to drive the transformation in the parasite could not be answered. Later, we tried to address this question while probing the downstream signaling of cAMP by phosphodiesterases (PDEs).

### 2.3. cAMP: A Major Upregulator of Antioxidant Genes of the Parasite

Normally, cytoprotection in eukaryotes depends on a number of molecular machineries, the most important of which are antioxidant enzymes. *Leishmania* has unique antioxidant machinery devoid of catalase and glutathione peroxidase. In most eukaryotic systems four enzymes have been implicated in antioxidant defense, namely, catalase, glutathione peroxidase, superoxide dismutase (SOD), and peroxidoxins (PXN). In *Leishmania*, instead of glutathione, trypanothione, a unique redox cycling glutathione-spermidine conjugate, is present, which in concert with trypanothione reductase (TR) maintains the intracellular reducing environment and resistance to reactive oxygen species (ROS). From genome analysis we know that at least 2 Sods and 3 different Pxns are present in *Leishmania,* of which Pxn1 is found to be differentially expressed and active against both ROS and reactive nitrogen intermediates (RNIs). A direct correlation between these antioxidant gene expression and intracellular cAMP response could be suggested from observations at both mRNA and protein levels of the genes, namely LdPxn1, LdSodA, and LdTr. They were all elevated by positive modulation of cAMP as well as on exposure to differential condition. Such upregulation of antioxidant genes of the parasite appears to be essential for induction of stress-resistance response of the parasite [19].

### 2.4. The Regulation of Leishmanial Adenylate Cyclases towards Positive Modulation of cAMP

Only very few publications have addressed adenylate cyclases in *Leishmania* for the last 20 years. Reports suggested that there are more than 10 adenylyl cyclases in this parasite. This surprisingly high number of different adenylyl cyclases might be related to any other peculiarity of the parasite. Interestingly, there is no report of any G proteins in the parasite, and therefore, possible functions of adenylate cyclases are yet to be deciphered. Previously, two receptor adenylate cyclases from *L. donovani *(LdracA and LdracB) were analyzed which form part of a cluster of five similar genes. They were observed to be developmentally regulated with their expression in promastigote stage and not in amastigote stage [21]. Since cAMP level was observed to be modulated during transformation from promastigote to amastigote stage, experiments were carried with these two isoforms. Interestingly, LdRACA knocked-down cells showed significantly decreased intracellular cAMP levels after exposure to differentiation condition starting from 30 minutes which decreased maximally after 2 hours of stress compared to uninduced set. Stress-unexposed parasites also showed decrease in cAMP levels in tetracycline-induced LdRACA knocked-down cells. LdRACB knocked-down cells showed little decrease in intracellular cAMP levels by tetracycline induction in both normal and 1 hourour stress exposure. This indicates towards the fact that LdRACA might be primarily responsible for modulation of cAMP level during stress (personal communications).

### 2.5. Receptor Adenylyl Cyclase Control in *Leishmania*: Probable Role of Pyrophosphate Pool and Pyrophosphatases

Since *Leishmania* lacks G-proteins, it was important to seek what provides a stringent control to the receptor adenylyl cyclases so that the strict local confinement of a cAMP signal, crucial for allowing local effect to occur, could be maintained. Many lower organisms were observed to have the total inorganic pyrophosphate pool (PPi) and polyphosphate pool (polyP) as environmental sensors. In *Leishmania*, control of adenylyl cyclases might be brought about by a further peculiarity of the parasite, that is, their high concentration of cytoplasmic PPi. This high concentration might effectively block cAMP synthesis via product inhibition of the adenylate cyclase reaction, the products being cAMP and PPi. Experiments were, therefore, designed to observe whether such PPi pool generates a negative feedback to receptor adenylyl cyclases towards formation of cAMP. The total PPi pool in log phase promastigote was found to be quite high (milimolar range), but it was interesting to note that differentiation condition exposure decreased the total PPi pool significantly by 1 hour. Modulation of PPi level might be largely due to the hydrolyzing enzyme, pyrophosphatase. *Leishmania *genome showed the existence of 3 different pyrophosphatases, namely, putative vacuolar type proton translocating pyrophosphatase (V-H^+^ ppase), soluble acidocalcisomal pyrophosphatase (LdVSP1), and putative inorganic pyrophosphatase (Ioppase). V-H^+^ ppase is known to be associated with acidocalcisomal membrane whereas vsp1 is soluble acidocalcisomal form and ioppase is of cytosolic localization. Expressions of all these pyrophosphatases were observed in both cytoplasmic and membrane fractions of *L. donovani *promastigotes by Western blot with antibodies raised against each of them by the administration of custom peptide in rabbit. V-H^+^ ppase was found to be predominantly present in membrane fraction of both normal and stress- (37°C and pH 5.5) exposed parasites. Its expression was not altered by the duration of stress exposure. Ldvsp1, on the other hand, was found to be present mainly in the cytoplasmic fraction of normal promastigotes. However, upon stress exposure, its expression was gradually enhanced in the membrane fraction with a maximum expression at 2 h after stress exposure with a plateau after 4 h. Further exposure did not alter LdVSP1 expression level (personal communications). The putatively cytosolic inorganic pyrophosphatase (ioppase) was mainly detected in the cytoplasmic fraction with its expression unaltered after stress exposure. Expression pattern of LdVSP1 could provide some clue of its control on PPi pool.

#### 2.5.1. Soluble Acidocalcisomal Pyrophosphatase: The Controller of Pyrophosphate Level in the Parasite during Stress

Relative expression and localization of the different pyrophosphatase proteins were then assessed in *L. donovani* promastigotes after stress exposure for various time periods by indirect immune fluorescence using antibodies raised against respective pyrophosphatases. As evidenced from immunofluoroscence, inorganic pyrophosphatase (ioppase) was cytosolic in both normal and stress-exposed cells. Neither did it colocalize with the acidocalcisome marker, nor it was found to be associated with any kind of membrane structure after stress exposure. The acidocalcisomal enzyme was found to be diminished significantly with time. LdVSP1 is primarily localized in vesicle-like structures of various sizes in normal unexposed promastigotes. Such cellular organization is typical of acidocalcisome distribution [22] and LdVSP1 seemed to be associated with parasite acidocalcisome in normal circumstances. Ldvsp1 was found to be colocalized with acidocalcisome marker (DND lysotraker green) in normal promastigotes, but after stress exposure for various time periods, the relative expression of LdVSP1 increased significantly. Moreover, after stress exposure, most of the LdVSP1 were localized near membrane structures. Acidocalcisomes was observed to move near membrane vicinity after 30 min of stress exposure and they could not be traced by its marker after 1 hour of stress. On the other hand, the V-H^+^ ppase was found to be colocalized with acidocalcisomal marker in both normal and stressed conditions and V-H^+^ ppase being a membrane bound acidocalcisomal pyrophosphatase could not be visualized after 1 hour of stress. These observations point towards the fact that the soluble acidocalcisomal LdVSP1 moves towards membrane vicinity by change in acidocalcisome biogenesis and function during stress (personal communications).

#### 2.5.2. Possible Role of Pyrophosphate in Leishmanial Adenylyl Cyclase Function

During stress, PPi pool was observed to get modulated, and interestingly, Ldvsp1 was found to be located near membrane vicinity after stress exposure, where leishmanial adenylyl cyclases (LdRACA and LdRACB) reside. To fully understand the regulation of leishmanial receptor adenylyl cyclases, experiment was carried to observe whether leishmanial adenylyl cyclases interact with LdVSP1 during stress. Observation indicated that LdVSP1 could interact with LdRACA but not with LdRACB during stress exposure (personal communications). This indicated that one part of cAMP regulation might have been contributed by pyrophosphatase enzymes along with the total inorganic pyrophosphate pool modulating the function of leishmanial adenylyl cyclases. The high concentration of PPi might effectively block cAMP synthesis via product inhibition of adenylyl cyclase reaction, the products of which are cAMP and PPi. These observations agree well with the previous speculation that LdRAC enzymes might have their catalytic domains stuck in a soup of PPi, being so strongly downregulated that they need activation which our study showed to be the action of soluble acidocalcisomal pyrophosphatase LdVSP1 (personal communication).

### 2.6. Downregulation of Intracellular cAMP by Cytosolic Phosphodiesterase (PDE)

Since it could be speculated that intracellular cAMP pool is regulated by adenylate cyclase (AC) with the help of enzymes like pyrophosphatases, it was exigent to observe another part of cAMP regulation by phosphodiesterases (PDE) that hydrolyze cAMP to 5′-AMP or cGMP to 5′-GMP. Since PDE activity is contributed by several families of PDE of which some might be located in the immediate vicinity of LdRAC, we would discuss the concrete information availed by us studying the leishmanial phosphodiesterases. Depending on the catalytic properties, PDEs are classified into 3 different categories, namely, class I, class II, and class III. 21 genes for PDE have been identified in mammals and several in *Drosophila* and *Dictyostellium*. Though, several class I isoforms have been identified in *T. bruci* and *T. cruzi* and PDE activity was previously reported in *Leishmania*, only very recently 2 PDEs have been cloned from *L. major* [23]. Since there is a large variety of PDEs in this lower eukaryote, some precise regulatory mechanism of intracellular cAMP must be maintained by them during the differentiation of the parasites. Among 4 different leishmanial phosphodiesterases (PDEA, PDEB1 and PDEB2, PDEC and PDED), PDEB and PDEC are predominantly membrane bound whereas PDEA and PDED are cytosolic. These PDEs might be a controlling factor for the differentiation of the parasites as the cytosolic PDE activity decreased during stage differentiation whereas the membrane bound PDE activity remained unaltered [24]. We extensively studied different PDEs of *L. donovani* and inferred that LdPDEA is differentially expressed and decrease of cytosolic activity is due to PDEA downregulation. Kinetic analysis showed detectable reduction of PDE expression 6 hours after exposure to differentiation condition and this was supported by immunofluorescence analysis [24]. We then tried to characterize PDEA by cloning the ORF in PET16b vector and expressed it bacterially. Enzyme kinetics showed a Km of 166.66 *μ*M for cAMP with no activity against cGMP. It was found to be a typical class 1 metal-dependent PDE (Ca^2+^-calmodulin independent and Mg^2+^-dependent). The mammalian PDE inhibitors could cause inhibition of this leishmanial PDE at very high concentrations barring 2 inhibitors (dipyridamole and trequinsin) proving it to be somewhat different from the mammalian counterpart [24]. 

#### 2.6.1. cAMP-Dependent PDEA: A Possible Target for Controlling Anti-Oxidant Machinery of the Parasite

In order to look into the functional significance of LdPDEA we then silenced the gene using tetracycline-inducible knock-down system [24]. When we used inhibitors of PDE, parasites showed enhanced viability against peroxide and peroxynitrite. Further, inhibition by pharmacologic inhibitors or knocking down PDEA caused enhanced peroxide degradation in the parasite. Peroxide neutralization in *Leishmania *is done by peroxidase as it lacks functional catalase. Since glutathione (GSH) is absent in *Leishmania,* peroxide action is mainly based on trypanothione (TSH), a glutathione-spermidine conjugate. Trypanothione is biosynthesized from arginine by arginase, ornithine decarboxylase (ODC), and other enzymes, which convert it to spermidine. It then conjugates with GSH. First we checked the availability of precursors like arginine and ornithine and found that the expression of arginine and ornithine transporter was not affected by PDEA inhibiton. Functional arginine and ornithine transport was also not affected [24]. But when we checked the expression of the enzymes for biosynthesis like arginase and ODC, we found that the expression of both these enzymes was increased under PDEA-inhibited condition suggesting thereby that PDEA inhibition might have caused increased TSH synthesis. But when we analyzed total thiol or intracellular TSH content, there was not much alteration [24]. We, therefore, wanted to check whether utilization of TSH pool was affected by PDEA inhibition. Normally, TSH pool is utilized in the parasite either by ribonucleotide reductase for DNA replication or by peroxidoxin and ascorbate peroxidase for peroxide degradation. In PDEA-inhibited parasites, expressions of all the enzymes which drive towards peroxide degradation like peroxidoxin and ascorbate peroxidase were elevated. Even the expression of intermediate electron shuttler like tryparedoxin was observed to get upregulated, which points to the fact that downregulation of PDEA may be needed for shifting the bias of TSH pool utilization toward antioxidant defense [24].

### 2.7. Downstream Effector of cAMP in *Leishmania*: Protein Kinase A

Though cAMP-dependent protein Kinase (PKA) is well characterized in eukaryote world, very little information is available on this particular downstream effector of cAMP signaling in the parasite. Our observations clearly indicated that temperature and pH stress which is responsible for transformation of promastigote to amastigote can also induce the PKA activity along with increasing cAMP levels. Moreover, substrate level phosphorylation on serine and threonine residues also increased during temperature and pH stress and in the case of positive modulation of cAMP by cell permeable cAMP analogs. PKA exists as inactive R_2_C_2_ heterotetramer consisting of two catalytic and two cAMP-binding regulatory subunits. Binding of cAMP to the regulatory subunits releases the active catalytic subunits, which are then free to phosphorylate a broad range of substrates. Recently, two PKA catalytic subunits (PKAC) from *Leishmania* have been cloned, characterized, and found to be sensitive to mammalian PKA inhibitors [25, 26] suggesting that PKA exists in *Leishmania *and perhaps plays a regulatory role in the parasite. In spite of the failure of previous attempts to identify PKA holoenzyme from kinetoplastidae parasites [27], functional PKAC-PKAR holoenzyme has recently been identified in *T. cruzi* [28]. To decipher the intricate role of PKA, it was indeed of utmost importance to study not only the catalytic subunit of PKA but also the regulatory counterpart. We for the first time have identified and characterized a functional PKA regulatory subunit (PKAR) from *L. donovani *[29]. Moreover, this report further extends the knowledge of cAMP-mediated responses in *Leishmania* as LdPKAR from *L. donovani* seemed to modulate metacyclogenesis, the process where the promastigotes get converted to infective form through induction of autophagy. Since regulatory subunits of PKA (PKAR) spatially and functionally interact with m-TOR during autophagosome maturation and deletion of PKAR results in activation of m-TOR leading to autophagic deficiency in mammalian cells and tissues [30, 31], our observation suggesting the role of LdPKAR in autophagy was really in line with the previous observations in the mammalian cells.

#### 2.7.1. Cloning and Characterization of a Regulatory Subunit of Protein Kinase A

The LdPKAR gene-encoding sequence was successfully cloned into the bacterial expression vector pET16b and expressed in *E. coli* BL21 (DE3) *pLysS* host. The fusion protein with an N- hexahistidine-tag was purified under nondenaturing conditions using Ni-NTA columns. It was found to be a single copy gene. The ORF of LdPKAR encodes a 502 amino acid polypeptide of molecular weight of 58.5 kDa. Comparison of protein sequences with *Trypanosoma* showed extensive identity with two of them, *T. brucei* (60%) and *T. cruzi* (66.1%), and showed 32.1% homology with bovine PKAR1-*α*. The N-terminal moiety of LdPKAR1 is longer than that of mammalian or *S. cerevisiae* PKARs and bears no identifiable functional domain. In analogy to other type 1 regulatory subunits, residues 133–137 and 203–207 probably represent the candidate pseudosubstrate sequences required for interaction and inhibition of PKA catalytic domain. Apart from this, residues 244–355 and 364–476 form the cyclic nucleotide binding domains A and B, respectively, which are composed of a number of conserved residues fitting the structural mode of bovine regulatory subunit PKAR1-*α* [32]. LdPKAR1 was regulated throughout the growth and differentiation cycle of the parasite as it is elevated in late stationary stage significantly compared to log phase promastigotes. Moreover, indirect immunofluorescence analysis in stationary phase promastigotes using polyclonal antibody raised against LdPKAR1 suggested it to be a predominantly cytosolic protein. The functional activation of PKA depends on the extent of dissociation of the catalytic and regulatory subunits. *Leishmania* reportedly encodes two functional PKACs [26], and therefore, each of these PKACs was tested for interaction with LdPKAR. LdPKAR interacted normally with LdPKAC1 and LdPKAC2 although LdPKAC2 interaction seemed to be weaker than LdPKAC1.

#### 2.7.2. Role of LdPKAR on Metacyclogenesis

LdPKAR expression was found to be increased in late stationary phase promastigotes, a condition metabolically similar to metacyclic promastigotes. In line with this observation, we found that the expression of LdPKAR significantly increases under starvation condition, a trigger for metacyclogenesis. Moreover, its overexpression could induce the onset of metacyclogenesis in the cells. Several properties like morphology, agglutination to PNA, increased expression of soluble acidocalcisomal pyrophosphatase (VSP1), sensitivity to human serum, and macrophage infectivity which distinguish metacyclic promastigotes from procyclic ones were all assessed to observe the role of LdPKAR on metacyclogenesis. Observations confirmed a definite role of LdPKAR on metacyclogenesis. Moreover, LdPKAR-over expressing cells were also found to be more efficient in surviving within IFN-*γ*-activated macrophages compared to wild-type parasites indicating greater infectivity of LdPKAR-over-expressing cells. LdPKAR appeared to have a role in the metacyclogenesis of *L. donovani* [29].

#### 2.7.3. Association of LdPKAR with Induction of Autophagy

PKA activity serves as a regulator of autophagy in a number of mammalian cell lines and such regulation seems to be evolutionarily conserved as autophagy is negatively regulated by Ras/PKA pathway in *S. cerevisiae* [33]. LdPKAR over-expression could also induce autophagy. Monodansyl cadaverine (MDC), an autoflurosescent marker that specifically labels autophagic vacuoles, was found to increase in LdPKAR-over-expressing starved cells. Possibly there was induction of autophagy in over expressed cells in starvation condition. *L. major* encodes a single copy ATG8 gene (LmjATG8) and fluorescent tracking of ATG8 entails autophagy monitoring in *Leishmania* parasites as efficiently as in mammalian and yeast cells [34]. Percentage of cells with ATG8-positive structures enumerated by using anti-LmjATG8 antibody was significantly higher in LdPKAR-over expressed cells compared to empty vector-bearing cells at 2 hours after starvation. ATG8-positive autophagosome formation in LdPKAR-over expressing cells could be prevented by addition of 3-methyl adenine and wortmanin, inhibitors of autopghagosome formation [35]. Ultrastructural analysis also showed more structures like autophagosomes and autophagolysomes in LdPKAR-over-expressing cells compared to cells bearing empty vectors at 2 h after starvation. These observations suggested that induction of metacyclogenesis by LdPKAR over expression might be due to induction of autophagy in *L. donovani* promastigotes. The significance of these observations with respect to cAMP signaling of the parasite for establishment of its infectivity seemed quite clear but further studies are required to decipher the intricate roles of all the components of leishmanial PKA [33].

## 3. Synopsis

**Figure 2:**
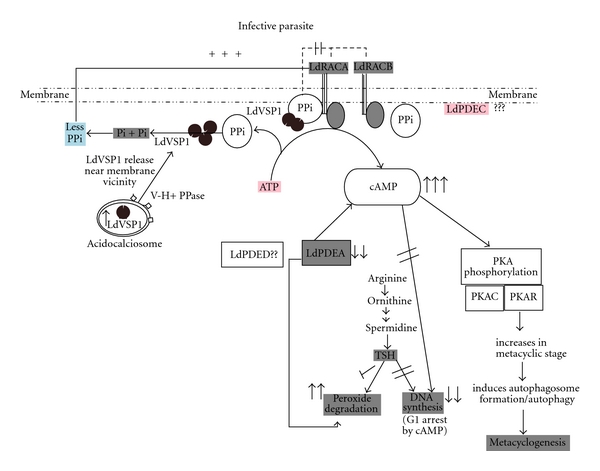
Model for comprehensive cAMP signaling in *Leishmania* parasites. Receptor adenylate cyclase A (LdRACA) and receptor adenylate cyclase B (LdRACB) are G-protein independent membranae bound adenylate cyclases (AC). LdRACA primarily converts ATP to cAMP along with the formation of PPi. This PPi pool provides an inhibition to AC towards the formation of cAMP. During stress, the PPi pool is hydrolyzed by vacuolar acidocalcisomal soluble pyrophosphatase (LdVSP1) which is released by membrane disintegration of acidocalcisomes releasing the inhibition on LdRACA to produce more cAMP. The increased level of cAMP stalls the cell cycle of the parasite at G1 stage and also elevates the expression of antioxidant genes like peroxidoxin, superoxide dismutase and tryparedoxin peroxidase. cAMP also downregulates a stage specific cytosolic PDE, LdPDEA leading to peroxide degradation due to trypanothione (TSH) pool utilization bias towards peroxide degradation instead of DNA synthesis by ribonucleotide reductase which helps in the survival of the parasite in macrophages. Moreover, LdPKAR might have a role in the infective metacyclic stage of the parasite as it induces the formation of autophagosome and process of autophagy initiating metacyclogenesis.

We first showed that differentiation-coupled induction of resistance of *Leishmania* parasites to macrophage oxidative damage is associated with increased intracellular cAMP and cAMP-mediated response. Parasites having increased cAMP-response elements were more cytoprotective, having higher levels of antioxidant enzymes and having more free radical scavenging capacity. For comprehensive understanding of cAMP signaling, we then studied the cAMP synthesizing enzyme, adenylate cyclase, the degrading enzyme phosphodiesterase (PDE), the regulatory enzyme pyrophosphatase (PPase) and the functional enzyme, cAMP-dependent protein kinase (PKA). Of 10 different leishmanial receptor adenylate cyclases (LdRACs), two (LdRACA and LdRACB) are stage-specific and developmentally regulated. Silencing and other biochemical parameters showed that differentiation-coupled induction of cAMP is regulated by LdRACA. We are the first to clone and characterize all five isoforms of PDE from *Leishmania* and showed that the soluble cytosolic isoform, PDEA, is heavily downregulated as the parasite is differentiated from promastigotes to amastigotes. Knockingdown the enzyme as well as by using specific inhibitors, we found that PDEA-inhibited parasites have markedly higher peroxide degradative capacity. This increased peroxide degradation is not due to increased trypanothione (TSH) biosynthesis or transport; rather it is due to the shifting of TSH pool utilization bias toward peroxide degradation, that is, antioxidant defense. Since pyrophosphate, one of the reaction product of adenylate cycase, is related with functionality of receptor adenylate cyclase, we studied the enzyme providing stringent control for it, that is, pyrophatases. Of the three different phosphatases present in *Leishmania,* the soluble acidocalcisomal form, LdVSP1 was found to be the major isoform regulating cAMP level and peroxide neutralizing capacity. The study on Ldvsp1 further suggests the significance of its association with LdRACA in regulating the cAMP pool which perhaps triggers the differentiation-associated events that ultimately affect the infectivity of the parasite (Figure 2). We then wanted to determine the downstream effector molecules of cAMP-mediated events. In mammalian cells, there are a number of intracellular effectors of cAMP, most important of which is cAMP-dependent protein kinase (PKA). PKA exists as inactive R_2_C_2_ heterotetramer consisting of two catalytic and two cAMP-binding regulatory subunits. Binding of cAMP to the regulatory subunits releases the active catalytic subunits, which are then free to phosphorylate a broad range of substrates. PKA catalytic (PKAC) subunits have been cloned and characterized from different *Leishmania* species, but the regulatory subunit has not yet been characterized from any *Leishmania* species. We identified a regulatory subunit of PKA from *L. donovani* (LdPKAR), which is expressed in all life cycle stages. Its expression attained maximum level in stationary phase promastigotes which are biochemically similar to infective metacyclic promastigotes. Starvation condition, the trigger for metacyclogenesis in the parasite, elevates PKAR expression, and under starvation condition, promastigotes overexpressing PKAR attained metacyclic features earlier than normal cells. Furthermore, PKAR overexpression accelerates autophagy, a starvation-induced cytological event necessary for metacyclogenesis and amastigote formation (Figure 2). Conditional silencing of PKAR delays the induction of autophagy in the parasite. The study, for the first time, reports the identification of a functional cAMP-binding effector molecule from *L. donovani* that may modulate important cytological events affecting metacyclogenesis. Since no *bona fide* cAMP-binding protein of defined function has yet been identified in *Leishmania* or in any other kinetoplastidae, the biological significance and molecular mechanism behind cAMP signaling is still an open field to be explored.
